# Sequential and Mixed Genetic Algorithm and Learning Automata (SGALA, MGALA) for Feature Selection in QSAR

**Published:** 2017

**Authors:** Habib MotieGhader, Sajjad Gharaghani, Yosef Masoudi-Sobhanzadeh, Ali Masoudi-Nejad

**Affiliations:** a *Laboratory of Systems Biology and Bioinformatics (LBB), Institute of Biochemistry and Biophysics, University of Tehran, Tehran, Iran.*; b *Laboratory of Bioinformatics and Drug Design (LBD), Institute of Biochemistry and Biophysics, University of Tehran, Tehran, Iran.*

**Keywords:** QSAR, Feature Selection, Drug Design, Genetic Algorithm, Learning Automata

## Abstract

Feature selection is of great importance in Quantitative Structure-Activity Relationship (QSAR) analysis. This problem has been solved using some meta-heuristic algorithms such as GA, PSO, ACO and so on. In this work two novel hybrid meta-heuristic algorithms i.e. Sequential GA and LA (SGALA) and Mixed GA and LA (MGALA), which are based on Genetic algorithm and learning automata for QSAR feature selection are proposed. SGALA algorithm uses advantages of Genetic algorithm and Learning Automata sequentially and the MGALA algorithm uses advantages of Genetic Algorithm and Learning Automata simultaneously. We applied our proposed algorithms to select the minimum possible number of features from three different datasets and also we observed that the MGALA and SGALA algorithms had the best outcome independently and in average compared to other feature selection algorithms. Through comparison of our proposed algorithms, we deduced that the rate of convergence to optimal result in MGALA and SGALA algorithms were better than the rate of GA, ACO, PSO and LA algorithms. In the end, the results of GA, ACO, PSO, LA, SGALA, and MGALA algorithms were applied as the input of LS-SVR model and the results from LS-SVR models showed that the LS-SVR model had more predictive ability with the input from SGALA and MGALA algorithms than the input from all other mentioned algorithms. Therefore, the results have corroborated that not only is the predictive efficiency of proposed algorithms better, but their rate of convergence is also superior to the all other mentioned algorithms.

## Introduction

In machine learning and data mining field, feature selection is a dimensionality reduction technique (1). In model construction the feature selection methods select a subset of relevant features. In feature selection techniques the evaluation methods are divided into five types: filter, wrapper, embedded, hybrid, and ensemble (1). The goal of feature selection is to determine the most critical features mainly (descriptors) more than hundred descriptors (2). In this paper the wrapper type among feature selection methods is used. Feature selection problem is an NP-Hard problem and for solving this problem different meta-heuristic algorithms have been used. In QSAR modeling different feature selection algorithms have been proposed. In QSAR modeling each feature indicates a molecular property while it is a number that denotes the properties of molecules like molecular weight, solvent accessible surface or other molecular properties. In other words any feature is considered as a single number that explains an aspect of a molecule (2). Ant Colony Optimization (ACO) algorithm (3) has been used for modeling of anti-HIV-1 activities of 3-(3,5-dimethylbenzyl) Uracil derivatives using MLR, PLS, and SVM regressions. Particle Swarm Optimization (PSO) and genetic algorithm (4) have been used for modeling of imidazo[1,5-a]pyrido[3,2-e]pyrazines, inhibitors of phosphodiesterase 10A. Modified ant colony optimization algorithm (5) had been used for variable selection in QSAR modeling on cyclooxygenase inhibitors. Monte Carlo algorithm (6) had been used for QSAR modeling on aldose reductase inhibitors. Particle swarm optimization and genetic algorithm (7) have been used for QSAR modeling of peptide biological activity. In this work two novel hybrid meta-heuristic wrapper algorithms i.e. Sequential GA and LA (SGALA) and Mixed GA and LA (MGALA), which are based on Genetic algorithm and learning automata for feature selection in QSAR model are proposed. SGALA algorithm uses advantages of Genetic algorithm and Learning Automata sequentially and the MGALA algorithm uses advantages of Genetic Algorithm and Learning Automata simultaneously. For evaluation of selected features for our proposed algorithms the MLR classification technique was used. Our proposed algorithms were executed on three different datasets (Laufer *et al*.(8), Guha *et al.*(9) and Calm *et al.*(10)). For evaluation and assessment of our proposed algorithms we implemented our proposed algorithms along with GA, ACO, PSO, and LA algorithms in MATLAB environment. Through implementing and running all the algorithms with different datasets, it was observed that the rate of converging to optimal result in MGALA-MLR and SGALA algorithms are better than GA, ACO, PSO, and LA algorithms and also the rate of MGALA algorithm is even better than SGALA and all other algorithms. A very important difference between LA and GA is that the GA tries to find the most appropriate chromosome from the population, but in LA the position of action is very important and therefore by combining these two algorithms (MGALA) the rate of convergence is improved. Error values in MGALA and SGALA algorithms are smaller than GA, ACO, PSO, and LA algorithms and **R**^2 ^values in SGALA and MGALA algorithms are more than GA, ACO, PSO, and LA algorithms in most runs as well. 

*Material and method*

*Genetic Algorithm (GA)*

Among the bio-inspired optimization algorithms, the Genetic Algorithm (GA), an algorithm based on the principles of natural selection, is believed to be one of the best and the most efficient ones (11). GA is a random search optimization algorithm that simulates the natural evolutionary theory. To this aim, it applies a fitness function and modeled the data into some chromosomes as initial population(11, 12). In this algorithm, the search process starts from initial population and by applying two operators (mutation and crossover) on the chromosomes the algorithm tries to generate new population and move to the optimal point of the search space. In each step, the distance of each chromosome to the optimal solution is measured by fitness function. Consequently, Optimization is the most critical function of the Genetic Algorithm(11, 13).

*Learning Automata (LA)*

**Figure 1:**
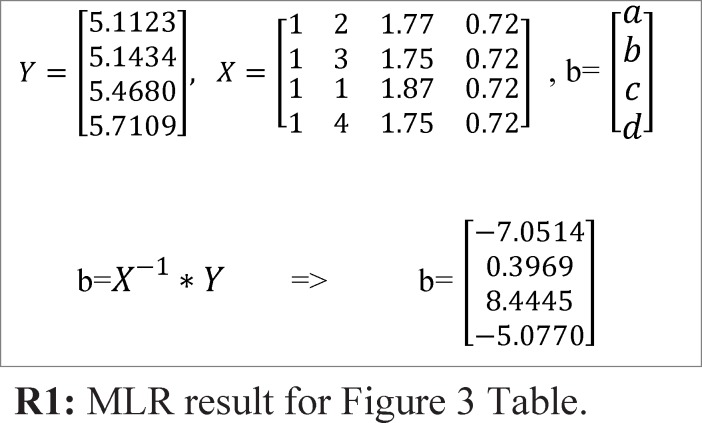
Learning automata connection with environment(16).

Learning Automata (LA) is perceived as an abstract model that selects an operation from a set of specific operations randomly. this algorithm employs the selected operation on the environment and informs the evaluated results by using a reinforcement signal (14). LA updates its interior states by means of selected operation and reinforcement signals. Then the algorithm selects the next operation in an iterative manner(15). The communication of LA and the environment is shown in Figure 1(16) .

The environment is shown by *E*={*α,β,c*} where *α*={*α*_1 _*,α*_2 _*,...,α*_r_} is a set of inputs, *β=*{ *β*_1 _*, β*_2 _*,... β*_r_} is a set of outputs, and *c={ c*_1 _,* c*_2 _,...* c*_r _*} *is penalty probabilities. When *β* is a set of binary, so the environment is a P type. In this kind of environment *β*_1_=1 is considered as penalty and *β*_2_=0 as reward (17, 18).

*Proposed Algorithms*

In this paper two novel hybrid algorithms for QSAR feature selection problem are proposed. These two new hybrid algorithms take advantage of both genetic algorithm and learning automata. In below sections the application of genetic algorithm and learning automata are described for feature selection in QSAR problem and then, the MGALA and SGALA algorithms are explained.

*Feature selection using GA*

**Figure 2:**
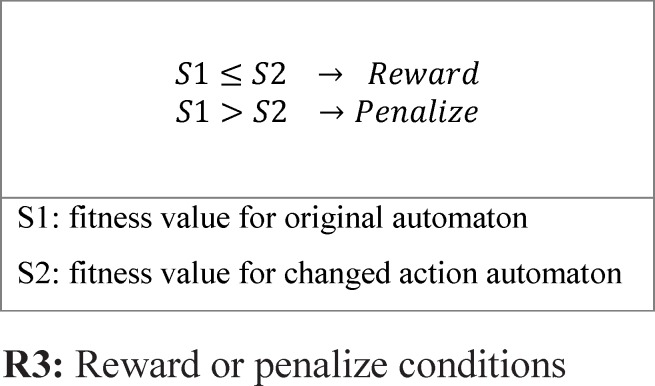
Proposed Genetic Algorithm flowchart

**Figure 3:**
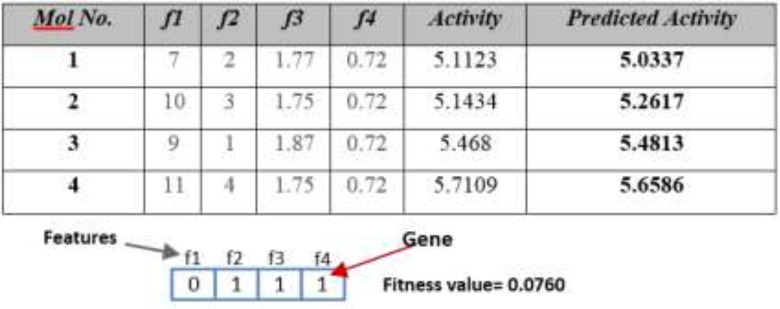
A sample of QSAR dataset and the relative random chromosome. Every feature, in dataset, is equal to a gene in chromosome. Gene value will be 1 if correspond feature is selected, and otherwise it will be 0

This algorithm tries to solve QSAR feature selection problem using Genetic Algorithm. The flowchart of this algorithm is depicted in Figure 2. At first this algorithm produces an initial population and then tries to converge to optimal result using genetic operations. Figure 3 shows a QSAR sample and corresponding chromosome for this algorithm.

The fitness function, crossover, and mutation operators are described in below sections:

*Fitness function*

To obtain the fitness value, first by using Multiple Linear Regression (MLR), the activity is predicted and after that by using Root Mean Square Error (RMSE) equation, the fitness value of each chromosome/automaton is calculated. For example, for sample Table and chromosome of Figure 3, the fitness value is determined using below steps:

*Step1*: Predicting activity using MLR. By using MLR, the activity values can be predicted. R1 relation shows the application of MLR for the sample demonstrated in Figure 3.

**Figure:**
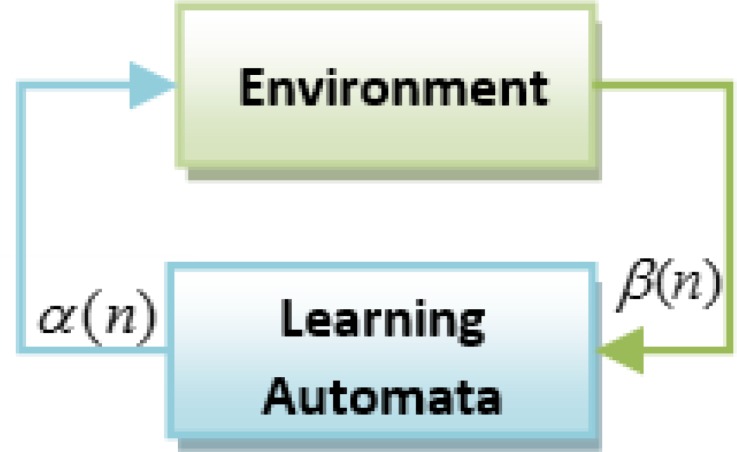


For this specific example, the predicted activity values could be calculated using below function: 

f=-7.0514+0.3969*f2+8.4445*f3 –5.0770*f4

*Step 2:* calculating chromosome fitness value using RMSE equation. After predicting activity values using MLR, the fitness value must be calculated using RMSE equation. R2 relation below shows the RMSE equation. In this function *n* is the number of sample molecules.

$\mathrm{Fitness}=F_{\mathrm{RMS}}=\sqrt[{2}]{\frac{\sum_{i=1}^{M}({{\mathrm{Ativity}}_{i}-{\mathrm{Predicted Activity}}_{i})}^{2}}{M}}$

**R2:** fitness value using RMS function

*Crossover operator*

**Figure 4:**
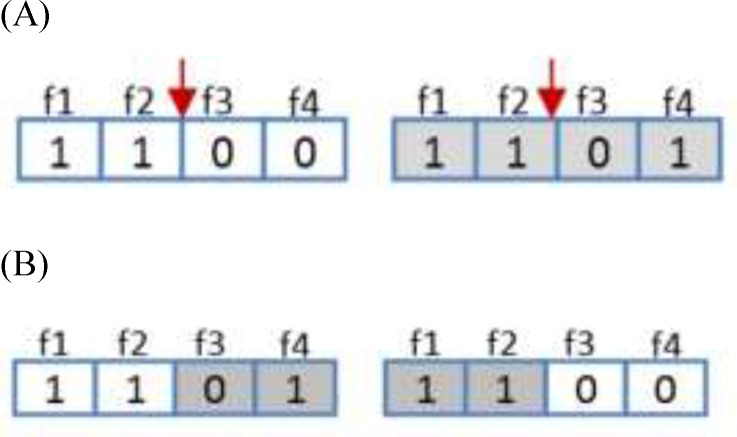
Crossover operator. (A) Two new chromosomes before crossover. (B) Two random chromosomes after crossover.

Regarding genetic algorithm, crossover operator  applied to modify the contents of chromosomes from one generation to the next ones. It is similar to the biological crossover process that the GA is based. The crossover procedure takes more than one parent solutions and generating the same number of child solutions from them. The crossover operator in this algorithm uses single point crossover. In this type of crossover two random chromosomes were selected and half of each chromosome was attached to the other chromosome and vice versa. This operator is depicted in Figure 4.

*Mutation operator*

**Figure 5:**
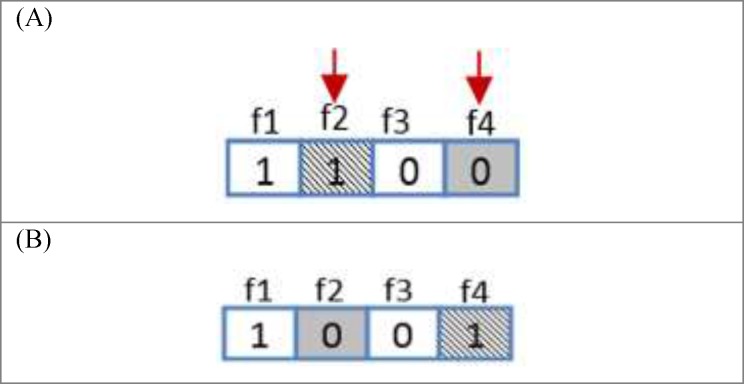
Mutation operator. (A) Resulted chromosome before mutation. (B) A random chromosome after mutation.

Similar to biological mutations, mutation operator is applied to sustain genetic variety from one generation of population to the next one. In mutation, the solution may alter completely from the previous solution. Therefore, GA can be improved to a better solution by using mutation. Mutation takes place in the course of evolution according to a user-defined mutation probability. The mutation operator type in this algorithm is order-based mutation. In this type of mutation two random genes are selected and the positions of them are swapped .This operator is illustrated in Figure 5.

*GA Termination*

In Genetic Algorithm there are some different conditions for termination of algorithm. In this paper at first the generation number is declared and then the algorithm executes according to this number.

*Feature selection using LA*

For a QSAR feature selection with *n* features, different 2^n^ states exist and if LA is applied to solving QSAR feature selection problem, LA must involve 2^n^ actions. In this article, the Object Migration Automata (OMA) method, proposed by Oommen and Ma, is utilized to reduce convergence speed. More precisely, the proposed algorithm utilizes Tsetlin automata, an OMA based algorithm, for solving QSAR selection problem (19).

**Figure 6:**
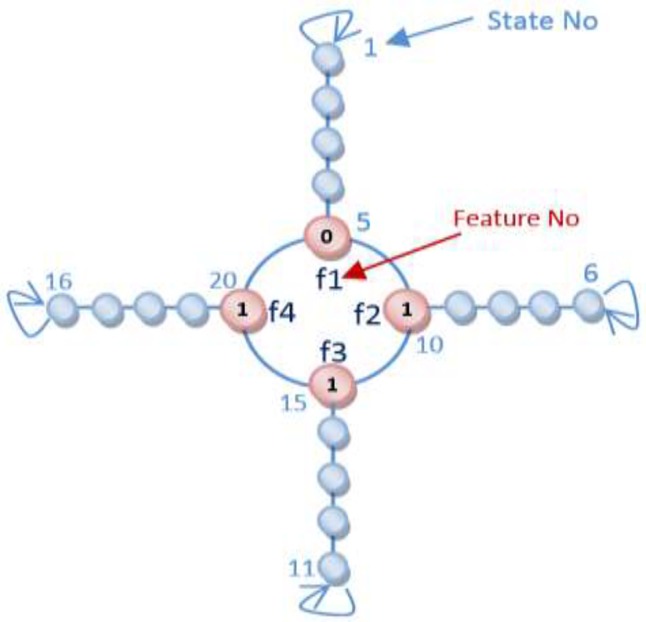
An equivalent automaton for chromosome in Figure 3.

**Figure 7:**
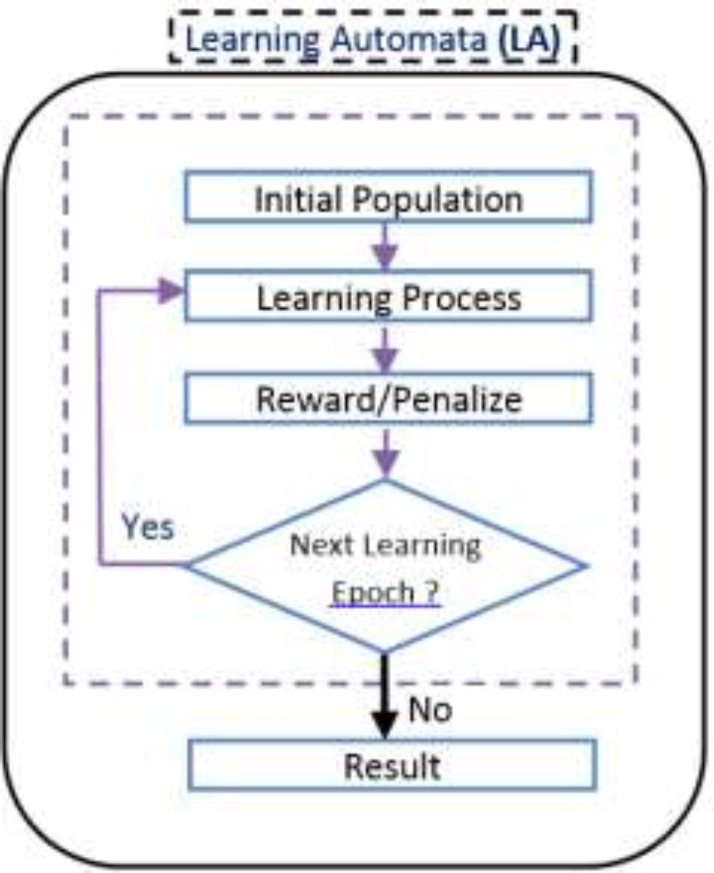
Proposed Learning Automata flowchart

In our proposed algorithms each chromosome is equal to an automaton and each gene is equivalent to an action of an automaton. The automaton illustrated in Figure 6 is equal to the chromosome which was brought in Figure 3. The flowchart of Learning Automata for solving this problem is depicted in Figure 7. In this algorithm at first the initial population consisting of some random automata is generated, and then by using LA method it tries to converge to the optimal result. By repeating the process of learning, the LA selects the suitable position of actions.

*Reward and penalize Operator*

**Figure 8:**
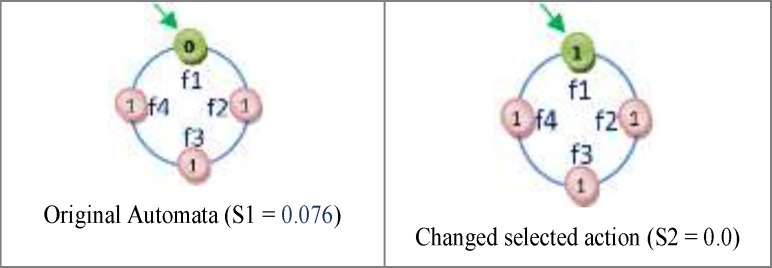
An example of Reward and Penalize relation

One of the important subjects in learning automata is reward and penalize operator. In this method in every epoch for every automaton an action is randomly selected and it is rewarded or penalized. At first the fitness value of automaton is calculated (suppose it is *S1*), after that if the selected action value is zero it changes to one and vice versa and then the fitness value of the altered automaton is calculated once again (suppose it is *S2*). Reward operator occurs when the value of *S1* is equal to or smaller than the value of *S2* and penalize operator occurs when the *S1* value is bigger than *S2* value (Figure 8). *R3 *relation shows the reward and penalizes.


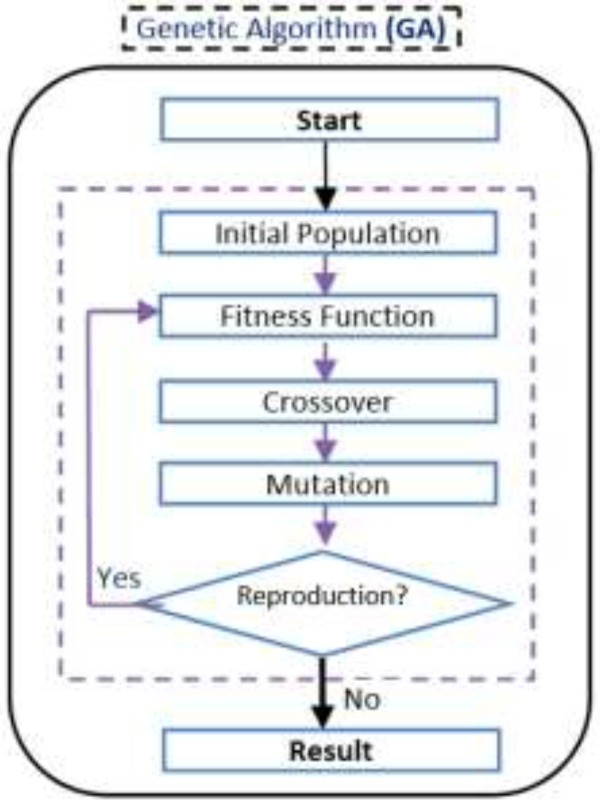


**Figure 9:**
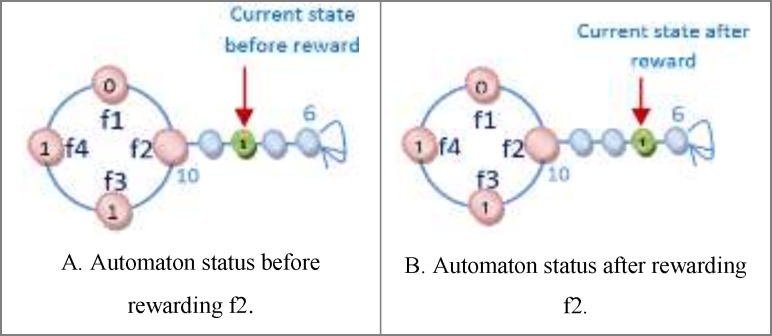
The trend of rewarding of feature f2

**Figure 10:**
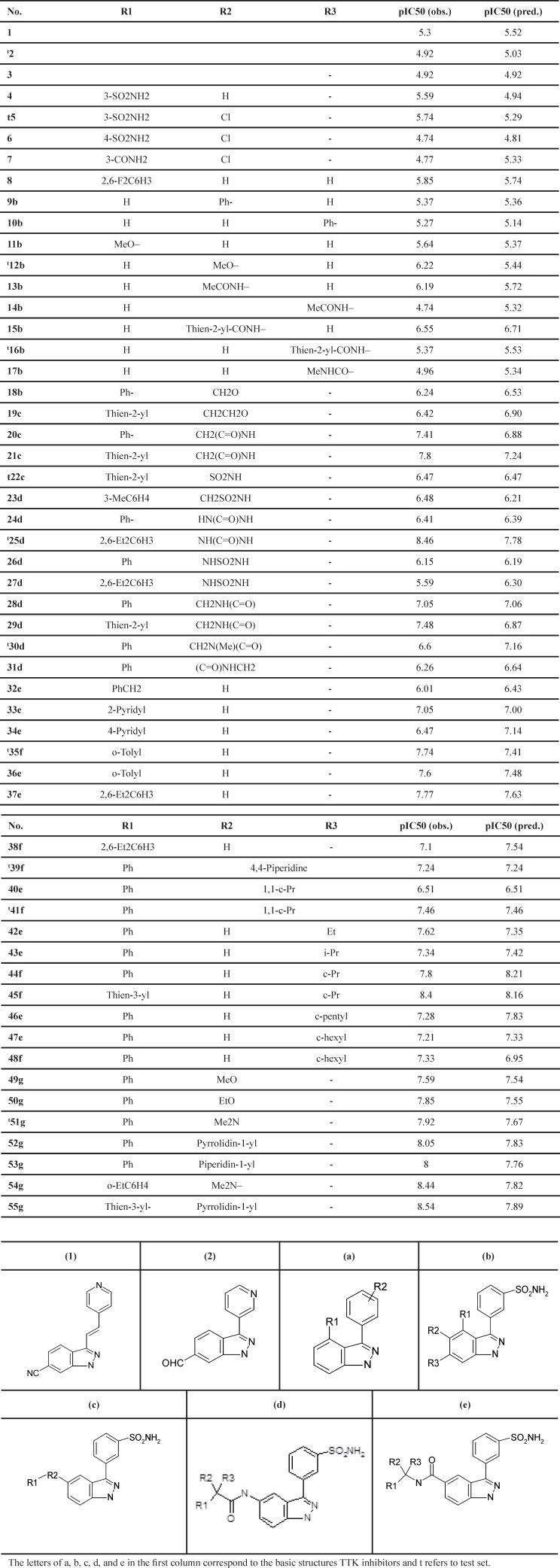
The trend of penalizing feature f2.

**Figure 11:**
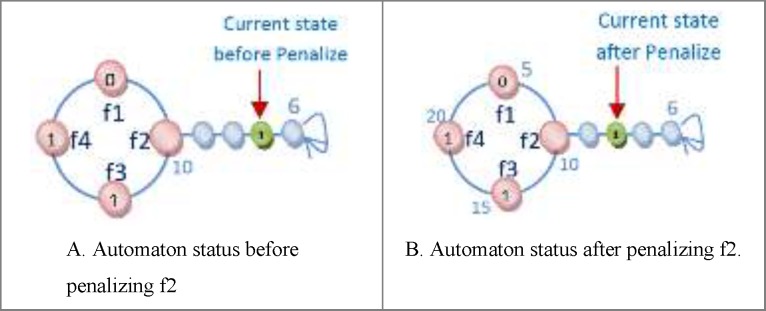
The trend of penalizing feature f1

For Figure 8 automaton, the selected action is penalized, because by changing the selected action value from zero to one its fitness value is minimized (the error is minimized). If the fitness value for the changed action is larger than original automaton fitness value, therefore the automaton is penalized. Figure 9 shows the reward operation and figures 10 and 11 show penalize operation.

Two possibilities are likely when penalizing an action:

(a) The action might occur in a position other than frontier position. In this case, penalizing makes it less important. The way the action of feature f2 is penalized, is shown in Figure 10.

(b) The action could occur in frontier position. In this case, provided that the value of action is *zero* it is turned into *one* and Vice versa. Figure 11 shows how feature f1 is penalized.

*Learning termination*

For termination of learning process there are different methods such as: predefined epoch number and obtained suitable result and etc. In this paper we use predefined epoch number and at first before the start of algorithm, the epoch number is defined and the algorithm repeats learning process using epoch number value. 

*Mixed GALA Algorithm (MGALA)*

GA tries to find the best chromosome in the population. In GA the location of genes, in chromosomes are random. 

**Figure 12:**
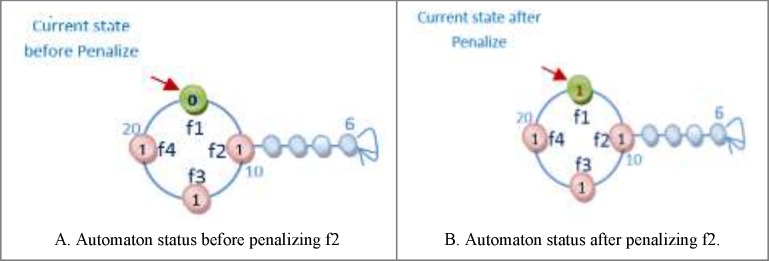
Proposed Mixed GA and LA flowchart

The optimal solution can be found in fewer generations if the position of the genes in the chromosomes discover optimally. Consequently, our algorithm tries to obtain the optimal solution in fewer generations utilizing the advantages of both GA and LA. In this algorithm the LA operator (reward/penalize) is added to GA. Generation number in GA and epoch number in LA in this algorithm are equivalent. The flowchart of this algorithm is shown in Figure 12. 

*Sequential GALA Algorithm (SGALA)*

**Figure 13:**
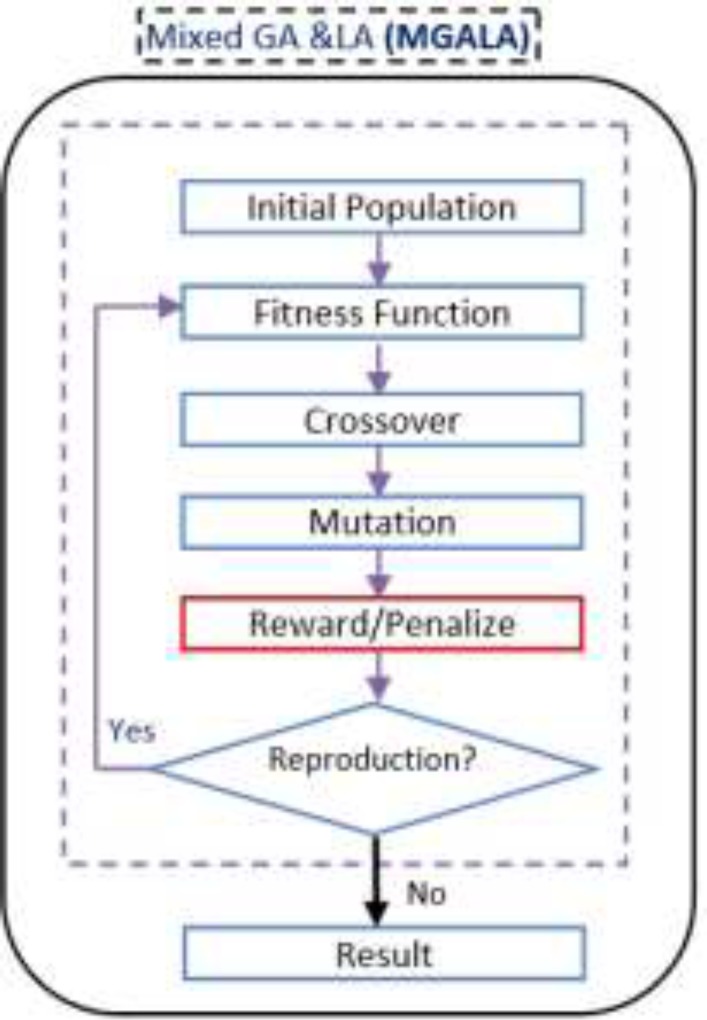
Proposed Sequential GA and LA flowchart

Another algorithm that we have proposed in this paper is SGALA. In this Algorithm at first the GA tries to converge to optimal result after a number of GA generations, the last population of GA is applied as the initial population of LA and next the LA tries to improve the last GA results. In this algorithm we could optimize the initial population of GA using less generation numbers and then by using LA the result could be improved. Generation number in GA and Epoch number in LA are distinct. The flowchart of this algorithm is exhibited in Figure 13. 

## Results

In this section we examine and evaluate the proposed algorithms with three different datasets. At first, the Laufer *et al.* (8) dataset was used for evaluation and examination of our proposed algorithms against GA, LA, PSO, and ACO algorithms and after that the best results of all algorithms were used as input for LS-SVR classifier model in which the differences of the results were reported. Secondly, two other datasets by Guha *et al*.(9) and Calm *et al.* (10) were used for the evaluation of proposed algorithms against GA, LA, PSO, and ACO algorithms. In this part only the rate of convergence to optimal result of the proposed algorithms and all other algorithms were compared and also the results of feature selection using the proposed algorithms and other algorithms were compared to each other.

*First experiment*

*Dataset*

The dataset used in the first experiment is derived from the Laufer *et al.* study (8). Table 1 shows the general chemical structures and the structural details of these compounds. This set contains the inhibitory activity values of N-(3-(3-sulfamoylphenyl)-1H-indazol-5-yl)-acetamides and carboxamides against TTK, reported in IC_50_ (µM). The IC50 values were converted into pIC_50_ (-log IC_50_) values. pIC_50_ is the relevant variable that distinguishes the biological parameters for the developed QSAR model. 

The inhibitory activities fall in the range 4.74 for inhibitors 6a and 14b to 8.54 for inhibitor 55d, with a mean value of 6.68. Table 1 depicts the basic structures of these inhibitors. The dataset was separated into two groups (training and test sets) using Y ranking method. The training and test sets consist of 44 and 11 inhibitors, respectively. The structures of molecules were drawn and optimized using HyperChem package (version 7.0) (20). The Semi-empirical AM1 algorithm with Polak–Ribiere was used as the optimization method until the root mean square gradient receives to 0.01 *kcal mol*^-1^ . The optimized geometries were used for the descriptor generation. 

The Dragon software is used to calculate the molecular descriptors (21). The MATLAB software version 7.6 and the free LS-SVM toolbox version 1.5 was used to derive all the LS-SVM models (22).

**Figure 14:**
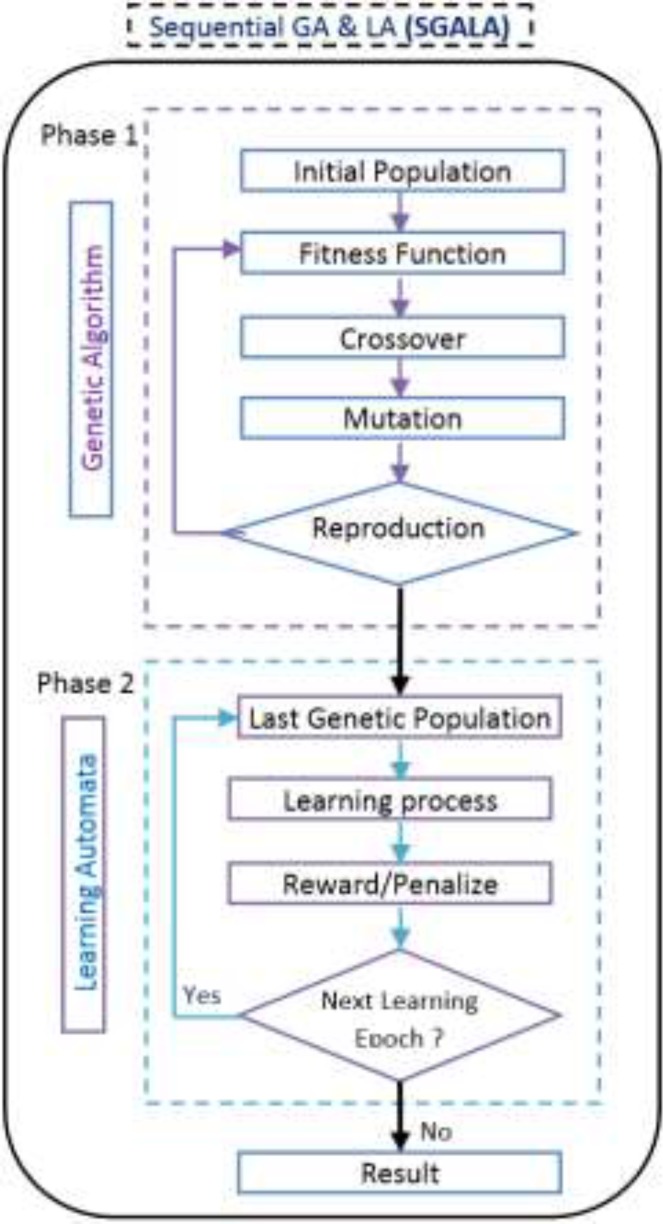
The variations of (A)$R^{2}$ and (B) RMSE for Table 3 results

**Figure 15:**
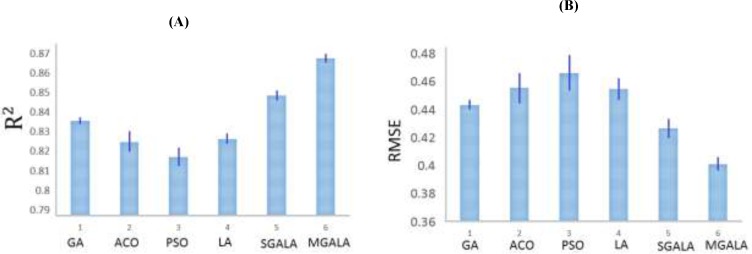
The average value of convergence process for the all mentioned algorithms on Laufer *et al*’s dataset. The number of generation is 100. The goal of the algorithms is minimizing RMSE value. MGALA and SGALA converge to minimum RMSE values than others

**Figure 16:**
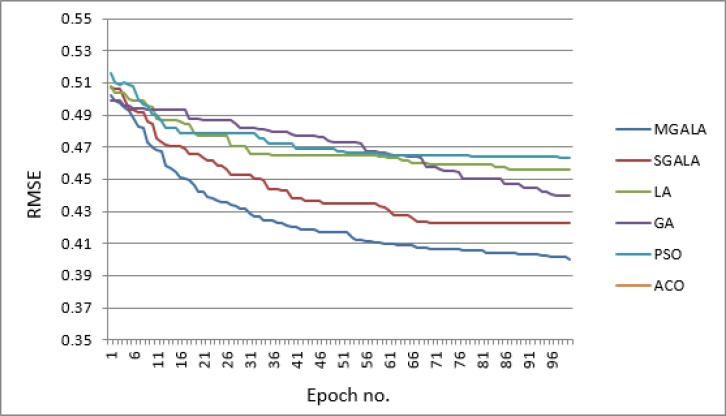
Plot of predicted pIC50 versus observed values using (A) GA-LS-SVR ($R^{2}\mathrm{test}=0.760$), (B) ACO-LS-SVR ($R^{2}\mathrm{test}=0.898$), (C) PSO-LS-SVR ($R^{2}\mathrm{test}=0.815$), (D) LA-LS-SVR ($R^{2}\mathrm{test}=0.786$), (E) SGALA-LS-SVR ($R^{2}\mathrm{test}=0.875$), (F) MGALA-LS-SVR ($R^{2}\mathrm{test}=0.770$) models

**Figure 17.**
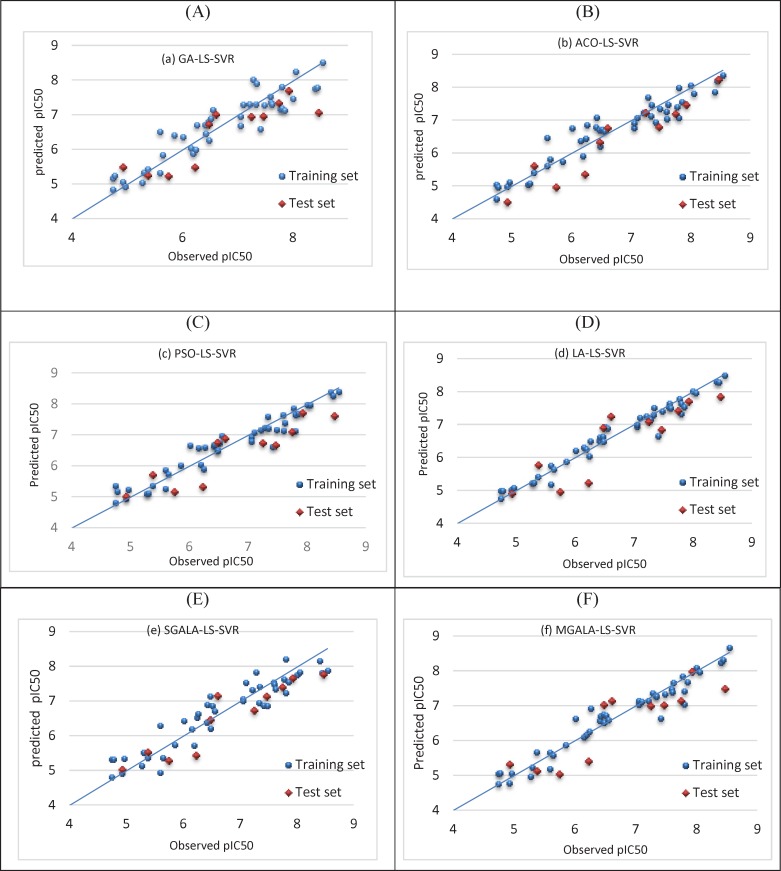
William’s plot of standardized residual *versus *leverage (h* = 0.54). (A) GA-LS-SVR, (B) ACO-LS-SVR, (C) PSO-LS-SVR, (D) LA-LS-SVR , (E) SGALA-LS-SVR, (F) MGALA-LS-SVR models

**Figure 18:**
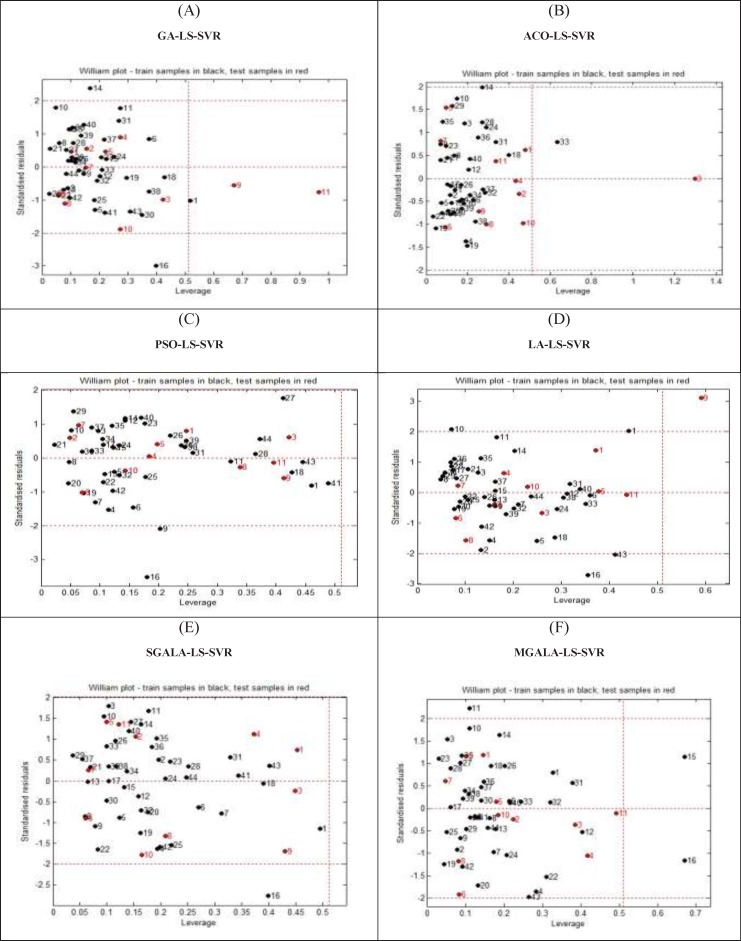
The results of variations of R2 for Table 6. (A) The R2 value for all the algorithms on *Guha et al’*s dataset. The MGALA and SGALA have the best R2 values than others have respectively. (B) The value for all algorithms on *Calm et al’*s dataset. MGALA and SGALA have the best R2 than others have respectively

**Figure 19:**
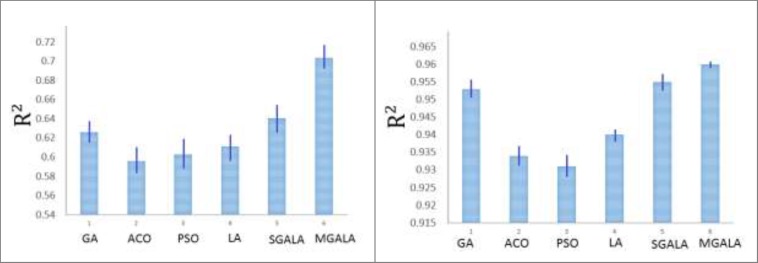
Average of convergence process for all algorithms for **(A)** Guha *et al.* and **(B)** Calm *et al.* datasets

**Table1:** Compounds list, observed, predicted pIC_50_ values, and Basic structures of TTK inhibitors.

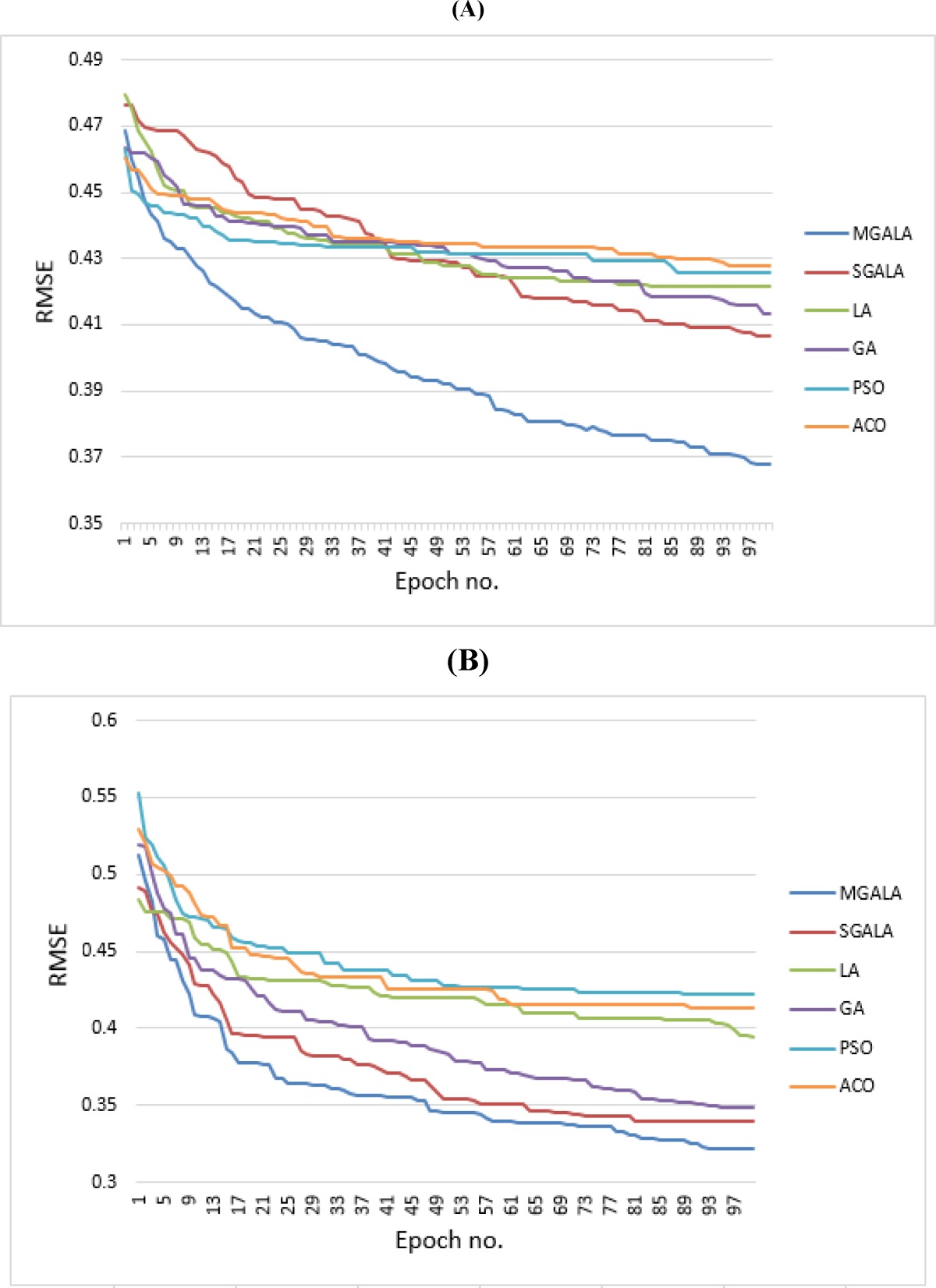

The letters of a, b, c, d, and e in the first column correspond to the basic structures TTK inhibitors and t refers to test set.

**Table 2:** Parameters of Algorithms

**Algorithm**	**GA**	**ACO(3, 38)**	**PSO(4, 39)**	**LA**	**SGALA**	**MGALA**
Initial Population	100	100	100	100	100	100
Generation	100	-	-	-	60	100
Epoch	-	100	100	100	40	-
Cross over	0.7	-	-	-	0.7	0.7
Mutation	0.3	-	-	-	0.3	0.3
Memory	-	-	-	3	3	3
Inertia weight(w)	-	-	0.8	-	-	-
Acceleration constants	-	-	1.5	-	-	-
Rho	-	0.7	-	-	-	-

**Table 3:** Results of algorithms for ten different Runs (Laufer *et al.* dataset

** Algorithm**	$R^{2}$ ** train**	**RMSE ** **train**	**Running time (second)**
**GA**			
Avg.	0.8351	0.44203	70.1
min	0.8274	0.4369	65
Max	0.839	0.4523	76
(Std.)	0.0035	0.0048	2.982
Best Result (feature names)	D/Dr05, MATS5m, MATS3v, ATS6e, SPAM, RDF035m, Mor08m, nCt		
**ACO**			
Avg.	0.825	0.454	93.144
min	0.800	0.435	79.65
Max	0.840	0.486	106.5
(Std.)	0.010	0.014	8.736
Best Result (feature names)	AMW, nCIR, RBN, DECC, BELp1, Mor17u, E3u, R1p+		
**PSO**			
Avg.	0.81796	0.46421	4.75012
min	0.8001	0.4255	4.2001
Max	0.8473	0.4868	5.721
(Std.)	0.0137	0.0178	0.4568
Best Result (feature names)	RDF095m, C-008, RBN, ISH, SPAM, GATS6e, MATS6e, nCaH		
**LA**			
Avg.	0.8263	0.4535	134.3
min	0.814	0.438	118
Max	0.8382	0.4695	170
(Std.)	0.0071	0.0092	19.4784
Best Result (feature names)	BEHv1,MATS3m,SPAM,RDF095m, Mor03u , Mor03m, E1u, nCaH		
**SGALA**			
Avg.	0.8470	0.4256	80.4
min	0.8315	0.4074	72
Max	0.86	0.4469	89
(Std.)	0.0071	0.0099	5.5892
Best Result (feature names)	RBN, X1A, BIC4, GATS5v, RDF035m, E2m, HATS1u, H8m		
**MGALA**			
Avg.	0.8647	0.4003	118.4
min	0.8596	0.3868	111
Max	0.8737	0.4079	125
(Std.)	0.0040	0.0060	4.4581
Best Result (feature names)	RBN, PW3, SAM, RDF095m, RDF120m, nSO2, C-027,H-046		

**Table 4:** The statistical parameters of the GA-LS-SVR, ACO-LS-SVR, PSO-LS-SVR, LA-LS-SVR, SGALA-LS-SVR, and MGALA- LS-SVR models

**Parameters**	**γ**	**δ** **2**	**R** **2 ** **train**	**RMSE train**	**R** **2 ** **test**	**RMSE test**
GA- LS-SVR	468.323	1413.690	0.861	0.409	0.760	0.591
ACO- LS-SVR	74.4881	88.5071	0.9028	0.3440	0.8980	0.4842
PSO- LS-SVR	8.2448	23.6189	0.9290	0.2965	0.8147	0.5578
LA-LS-SVR	27.965	19.1907	0.964	0.210	0.786	0.545
SGALA-LS-SVR	119.877	465.674	0.880	0.381	0.875	0.443
MGALA-LS-SVR	1007.3	293.604	0.940	0.268	0.770	0.564

**Table 5:** The statistical parameters of the external test set for GA-LS-SVR, ACO-LS-SVR, PSO-LS-SVR, LA-LS-SVR, SGALA-LS-SVR, and MGALA-LS-SVR models

**Parameters **	**GA- LS-SVR**	**ACO- LS-SVR**	**PSO- LS-SVR**	**LA- LS-SVR**	**SGALA-LS-SVR**	**MGALA-LS-SVR**
$Q^{2}$	0.698	0.803	0.731	0.744	0.830	0.725
$r_{p}^{2}$	0.759	0.898	0.815	0.786	0.875	0.770
r0 p2	0.665	0.897	0.772	0.776	0.859	0.750
${\overset{\text{´}}{r}}_{0p}^{2}$	0.758	0.880	0.815	0.769	0.875	0.759
$\left\lfloor {(r}_{p}^{2}-r_{0p}^{2})/r_{p}^{2}\right\rfloor$	0.123	0.001	0.053	0.012	0.018	0.026
$\left\lfloor {(r}_{p}^{2}-r_{0p}^{2})/r_{p}^{2}\right\rfloor$	0.001	0.020	0.000	0.0201	0.000	0.014
$r_{m}^{2}$	0.526	0.870	0.646	0.709	0.764	0.661
${\overset{\text{´}}{r}}_{m}^{2}$	0.734	0.777	0.815	0.685	0.875	0.689
k	0.954	0.952	0.950	0.969	0.963	0.964
$\overset{\text{´}}{k}$	1.041	1.047	1.048	1.026	1.035	1.031

**Table 6:** Results of algorithms for ten different Runs (Guha *et al.* and Calm *et al.* datasets

	**Dataset ** **1**					**Dataset ** **2**								
	**Feature size**	**Mol no.**		**References**	**Selected features no.**	**Feature size**				**Mol no.**		**References**		**Selected features no.**
	320	79		(9)	12	115				45		(10)		7
	$R^{2}$ train	RMSE train	Running time (second)			$R^{2}$ train					RMSE train		Running time (second)	
GA														
Avg.	0.626	0.413	220.184				0.953				0.348		40.332	
min	0.616	0.401	189.797				0.946				0.283		36.935	
Max	0.647	0.419	291.226				0.969				0.376		48.873	
(Std.)	0.009	0.005	27.303				0.006				0.027		3.740	
Best Result (feature names)	MOLC#5, EMAX#1, MOMI#3, GRAV#3, CHDH#2, CHDH#3, SCDH#1, SAAA#1, SAAA#3, CHAA#2, ACHG#0						BEHm2, ATS1m, MATS1m, DISPe, RDF020u, E3s, HTp							
ACO														
Avg.	0.596	0.428	101.835			0.934					0.413		15.344	
min	0.564	0.415	86.5			0.923					0.379		12.196	
Max	0.613	0.446	116.54			0.945					0.448		26.201	
(Std.)	0.016	0.009	10.237			0.007					0.022		4.081	
Best Result (feature names)	MOLC#4, WTPT#2, WTPT#5, MDEC#12, MDEN#33, MREF#1, GRVH#3, NITR#5, FNSA#2, SADH#3, CHDH#3, FLEX#5					AMW, Me, X4v, IDDE, L3m, HTp, nROR								
PSO														
Avg.	0.603	0.425	7.057			0.931					0.422		5.728	
min	0.583	0.410	0.627			0.9164					0.3821		4.233	
Max	0.632	0.436	11.147			0.9445					0.4687		7.781	
(Std.)	0.018	0.010	2.662			0.008					0.027		1.127	
Best Result (feature names)	2SP2#1, CHAA#2, CHDH#2, WNSA#1, WTPT#4, PNSA#2, N2P#1, SADH#2, SADH#1, NITR#5, SURR#1, MOLC#3					R1u, nF, S2K, nROR, L3m, AMW, HTp								
LA														
Avg.	0.61146	0.4215	265.617			0.940					0.393		56.644	
min	0.5945	0.4022	215.16			0.934					0.372		44.469	
Max	0.646	0.430	307.315			0.947					0.413		74.579	
(Std.)	0.016	0.008	31.762			0.003					0.012		9.514	
Best Result (feature names)	V3CH#15, WTPT#4, MDEC#34, MDEO#12, MREF#1, EMIN#1, MOMI#1, VOL#150, homo#0, WPSA#1, FNHS#1, RNH#1					ATS2e, RPCG, DISPe, L3m, H0e, HTp, F082								
SGALA														
Avg.	0.6409	0.4051	270.678					0.955			0.340		61.969	
min	0.624	0.382	245.521					0.946			0.312		46.548	
Max	0.680	0.414	298.413					0.962			0.374		76.731	
(Std.)	0.017	0.009	18.779					0.005			0.019		9.553	
Best Result (feature names)	KAPA#2, KAPA#4, ALLP#1, ALLP#2, V4PC#12, N6CH#16, N7CH#20, NITR#5, FNSA#3, RNCG#1, SCDH#2, FNHS#1							IDDE, SHP2, DISPe, L3m, H0e, HTp, Hy						
MGALA														
Avg.	0.704	0.371	627.026			0.960					0.322		124.922	
min	0.684	0.352	555.923			0.958					0.302		89.526	
Max	0.727	0.390	709.474			0.965					0.329		150.749	
(Std.)	0.013	0.010	53.150			0.001					0.007		20.326	
Best Result (feature names)	KAPA#2, KAPA#4, ALLP#4, V5CH#17, S6CH#18, MOLC#1, SADH#2, CHDH#1, CHDH#3, SAAA#2, ACHG#0, SURR#5								GGI1, DISPe, RDF020u, E3s, H0e, HTp, Hy					

*Descriptor calculation and selection*

These descriptors were presented in a two dimensional data matrix whose rows and columns store the inhibitors and descriptors, respectively. Some preprocessing operations should be applied after calculating the molecular descriptors. Then the following procedure selects some of the important descriptors. At the beginning, those descriptors that remained constant for all the inhibitors were ignored. Variable pairs with a Pearson correlation coefficient larger than 0.80 were considered as inter-correlated. One of them was used to develop the feature selection model and the other one was ignored. After this process, totally 221 descriptors remained for further investigation.

Subsequently, the GA, ACO, PSO, LA, SGALA, and MGALA algorithms were used to select the most feasible descriptors from 221 remained descriptors which were related to the anti-cancer activity of inhibitors. In this paper for performance evaluating of algorithms we used MATLAB software environment. For all of the four algorithms we implemented code in MATLAB and then results of proposed algorithms in different runs were evaluated and compared with each other. All runs were performed on AMD Phenom II Quad-core 1.8 GHz and 4 Gb Ram. Our examinations are done on anti-Cancer datasets. This dataset has 55 molecule and 221 descriptors. So our proposed algorithms tries to find some features that minimize Error value or maximize R^2^ value. It is suggested that the number of samples should be 5 times larger than the number of features (23, 24). Therefore, maximum number of feature that our proposed algorithms try to find it is 20% of inhibitor numbers in the training set. The results of all the algorithms are explained in Tables 3. The mean R^2^ of SGALA and MGALA is higher than those of other algorithms and the mean RMSE value of our proposed algorithms is less than those of others. The properties of algorithms are demonstrated in Table 2. The variations of RMSE and R^2^ values for this Table are depicted in Figure 14.

In Figure 15, the process of convergence to optimal result for proposed algorithms and other algorithms is depicted. In this figure every result is the average of ten random executions. Itʹs evident that the MGALA and SGALA convergence rates are better than those of others and the convergence rate of MGALA algorithm is even better than SGALA algorithm. The final results of MGALA and SGALA are better than other algorithms.

*LS-SVR Model*

For the modeling studies we selected best runs from algorithms which present a good combination of R^2 ^and RMSE results***.*** To investigate relation between selected molecular descriptors and pIC_50_, we used Least Squares-Support Vector Regression (LS-SVR) as a non-linear feature mapping technique. In this model, the input vectors are the set of descriptors selected by feature selection algorithms. 

The radial basis function (RBF) was utilized as a kernel function, which represents the distribution of sample in the mapping space. RBF is a non-linear function and can reduce the computational complexity of training procedure (25). The next step was optimization of LS-SVR parameters, including regularization parameter (γ) and kernel parameter (δ^2^). 

The optimized values for the parameters were achieved from grid search method. As mentioned before it, all algorithms introduced in this paper have used LS_SVR model and RBF kernel function. Sigma value of RBF kernel function is effective in model generation. The Higher is sigma value, the more flat is Gaussian distribution; so the decision boundary is smoother. Lower sigma value of RBF kernel function will make sharper the Gaussian distribution and also the decision boundary will be more flexible (26). The best value of sigma which enhances the model performance is achieved using grid search method. The sigma values of all mentioned models are inserted in Table 4. The sigma values of SGALA-LS-SVR and MGALA-Ls-SVR models are proper than other models so their Gaussian distribution is not sharp or smooth.

Besides sigma parameter which has influence on model regression, the gamma regulation value minimizes training error and model complexity. Over-fitting will occur if values of sigma and gamma are enhanced (27, 28). Therefore, these values must be carefully selected. We observe that the sigma and gamma values of SGALA-LS-SVR and MGALA-Ls-SVR models are not maximum value simultaneously in Table.4. The parameter values inserted in Table.5 show that proposed models are acceptable. 

The significance and predictability of the constructed model was evaluated using the external set and the statistical parameters were recommended by Trospsha (29, 30) and Roy (31). They suggested a number of criteria that assess the predictive ability of a QSAR model; 

 R4: $Q^{2}$> 0.5

 R5: $r_{p}^{2}$ > 0.6

 R6: $\left\lfloor {(r}_{p}^{2}-r_{0p}^{2})/r_{p}^{2}\right\rfloor$ or $\left\lfloor {(r}_{p}^{2}-{\overset{\text{´}}{r}}_{0p}^{2})/r_{p}^{2}\right\rfloor <0.1$

 R7: 0.85 ≤ k ≤ 1.15 or 0.85 ≤ $\overset{\text{´}}{k}$≤ 1.15

R8:${r_{m}^{2}=r}^{2}\left(1-\left|\sqrt[{2}]{r^{2}-}r_{0}^{2}\right|\right)$ and ${\overset{\text{´}}{r}}_{m}^{2}>0.5$

The statistical parameters of the GA- LS-SVR, ACO-LS-SVR, PSO-LS-SVR, LA-LS-SVR, SGALA-LS-SVR, and MGALA-LS-SVR models were compared. The results are given in Table 5. All models have *Q*^2^ values larger than 0.5 and *r*_p_^2^ values higher than 0.6.

The performance of all models was evaluated by plotting the predicted values of pIC_50_ against experimental values for the training and test sets. The results are shown in Figure 16. 

This figure shows that there is a good agreement between the observed activity and the predicted values.

*Applicability Domain assessment*

One of the crucial problems in QSAR modeling is the definition of its Applicability Domain (AD). There are different methods for obtaining applicability domain in QSAR models (32). One of the common methods is defining leverage values for every compound (33). In this work, the applicability domain is verified by the William’s plot. The applicability domain is settled inside a squared range within ±3 standard deviation and a leverage threshold$h^{*}$ (${\begin{array}{c} h \end{array}}^{*}=\frac{3p}{n}$ , where *p* is the number of model parameters and *n* is the number of compounds). In Laufer *et al.* dataset, the *p* and *n* values are 8 and 44 respectively. Therefore, in this dataset h^*^ value is 0.54. Figure 17 shows the applicability domain for all GA-LS-SVR, ACO-LS-SVR, PSO-LS-SVR, LA-LS-SVR, SGALA-LS-SVR, and MGALA-LS-SVR models. It can be seen from this Figure that the majority of compounds in the train and test datasets are inside the squared region. In SGALA-LS-SVR model, all of the train and test compounds are inside the squared region and therefore there is no outlier compound in this model. In MGALA-LS-SVR model, only two train compounds (19c and 20c) have more leverage value than h^*^ and all of the others are inside the squared area. A Matlab toolbox (version 1.0, Milano Chemometrics and QSAR Research Group) was used for all of the six mentioned QSAR models (34, 35) in order to evaluate and assess the applicability domain and William’s plots.

*Cross-Validation*

To assure the impartial comparison of the classification outputs and in order to avoid generating random results, this study applied a Leave-One-Out cross validation (LOOCV) methodology. Cross-validation is a statistical procedure that divides data into two segments for comparing and evaluating learning algorithms. One part is usually used to learn or train the model and the other is applied to validate the model (36)

*Second experiment*

In this section all of the mentioned algorithms are applied on Guha *et al.*(9) and Calm *et al.* (10) datasets. For all of the datasets, 80% of data were assumed as train data and next 20% data were assumed as test data. The LOOCV cross-validation method was used for classification results. Because the number of features that algorithms try to find is 20% of inhibitor numbers in the training set, therefore for Guha *et al.* dataset the number of selected features was 12 and for Calm *et al*. dataset were 7. The properties of algorithms are demonstrated in Table 6. The variations of R^2^ values for this Table are depicted in Figure 18.

In Figure 19 the process of converging to optimal result for proposed algorithms and other mentioned algorithms are depicted. In this figure every result is the average of ten random executions. Itʹs evident that the MGALA and SGALA converging rates are better than those of others and the converging rate of MGALA algorithm is even better than SGALA algorithm for Guha *et al*. and Calm *et al.* datasets. The final results of MGALA and SGALA are better than all other algorithms. 

## Discussion

Descriptor selection has been used with various algorithms on QSAR data. Using the same data is essential in order to compare algorithms. As mentioned in the manuscript, SGALA and MGALA algorithms are suggested for descriptor selection. We implemented PSO, GA, and ACO algorithms because these algorithms had been applied on different data not available now. The results of proposed algorithms have been compared with GA, PSO, and ACO algorithms. The acquired results are descripted as follow:

In reference (4), Goodarzi *et al* have proposed two GA and PSO algorithms using three different regression methods as: multiple linear Regression (MLR), Locally Weighted Regression based on Euclidean distance (LWRE), and Locally Weighted Regression based on Mahalanobis distance (LWRM). 

All of the algorithms had been implemented on “*imidazo[1,5-a]pyrido[3,2-e]pyrazines, inhibitors of phosphodiesterase 10A” *dataset with 46 train and 15 test compounds. In this paper reported *R*^2^_train_ values on PSO/MLR, GA/MLR, PSO/LWRE, PSO/LWRM, GA/LWRE, and GA/LWRM were 0.82, 0.85, 0.81, 0.81, 0.85, and 0.85 respectively. Also *R*^2^_test _reported values were 0.87, 0.79, 0.89, 0.87, 0.76, and 0.76 respectively. In another work (37), GA/MLR had been executed on *“imidazo[1,5-a]pyrazine derived ACK1 inhibitors” *dataset and the reported value for *R*^2^_train_ over 30 samples was 0.8. Ant colony optimization algorithm along with PLS, MLR, and SVM regressions had been executed on *“anti-HIV-1 activities of 3-(3,5-dimethylbenzyl)uracil derivatives”* datasets (3). In this research, produced *R*^2^_train_ values for ACO/MLR, ACO/SVM, and ACO/PLS on 34 compounds were 0.983, 0.991, and 0.983 respectively and* R*^2^_test_ values on 9 compounds were 0.942, 0.991, and 0.945 respectively. 

In our work GA/MLR, ACO/MLR, PSO/MLR, LA/MLR, SGALA/MLR, and MGALA/MLR have been executed on three different datasets. Our proposed SGALA/MLR and MGALA/MLR algorithms have produced better results than those of GA/MLR, ACO/MLR, and PSO/MLR algorithms . Therefore, it is expected that the results will be better by executing our proposed new algorithms on different QSAR datasets.

## Conclusion

In this paper two novel hybrid algorithms based on Learning Automata and Genetic Algorithm have been proposed for feature selection in QSAR. Through implementing and running all the algorithms with different datasets, it was observed that the rate of converging to optimal results in MGALA and SGALA algorithms are better than GA, ACO, PSO, LA algorithms and the rate of MGALA algorithm is better than SGALA and all other algorithms. A very important difference between LA and GA is that the GA tries to find the most appropriate chromosome from the population, but in LA the position of action is very important and therefore by combining these two algorithms (MGALA) the rate of converging is improved. Error value in MGALA and SGALA algorithms is smaller than GA, ACO, PSO, and LA algorithms and R^2^ value in SGALA and MGALA algorithms is more than GA, ACO, PSO, and LA algorithms in most runs as well. Different runs for all algorithms demonstrated that the produced results by MGALA algorithms are better than SGALA algorithm and SGALA algorithm is better than all GA, ACO, PSO, and LA algorithms. After selecting some features using GA, ACO, PSO, LA, SGALA, and MGALA algorithms, the output of algorithms was applied separately as the input of LS-SVR model. The results of GA-LS-SVR, ACO-LS-SVR, PSO-LS-SVR, LA-LS-SVR, SGALA-LS-SVR, and MGALA-LS-SVR models have proved that the SGALA-LS-SVR and MGALA-LS-SVR models are of high predictive ability and are able to fulfill all the criteria. These results have revealed that the performances of the SGALA-LS-SVR and MGALA-LS-SVR models are superior to those of GA- LS-SVR, ACO-LS-SVR, PSO-LS-SVR, and LA-LS-SVR models. 
